# Dysbiosis and Migraine Headaches in Adults With Celiac Disease

**DOI:** 10.7759/cureus.28346

**Published:** 2022-08-24

**Authors:** Hodan Qasim, Mohamed Nasr, Amad Mohammad, Mosab Hor, Ahmed M Baradeiya

**Affiliations:** 1 Internal Medicine, Alfaisal University College of Medicine, Riyadh, SAU; 2 Internal Medicine, Mansoura General Hospital, Mansoura, EGY; 3 Hematology and Oncology, Saint James School of Medicine, New York, USA; 4 Ophthalmology, Children Retina Institute, Los Angeles, USA; 5 Ophthalmology, Palestinian Medical Council, Ramallah, PSE; 6 Internal Medicine, Fresno Clinical Research Center, Fresno, USA

**Keywords:** gut-brain axis, celiac disease and microbiota, gluten and microbiota, microbiota and migraine, celiac disease and migraine

## Abstract

One of the most significant illnesses associated with gluten is celiac disease, which encompasses many conditions. It is generally recognized that neurological manifestations can occur either at the time of the disease onset or as the illness continues to develop. One of the main clinical presentations of celiac disease is headache, either in the form of migraine or in an unspecific form. Migraine pathophysiology is intricate and still poorly understood. Several mechanisms involving the gut-brain axis have been proposed to explain this association. These include the interaction of chronic inflammation with inflammatory and vasoactive mediators, the modulation of the intestinal immune environment of the microbiota, and the dysfunction of the autonomic nervous system. However, further research is required to fully comprehend the fundamental mechanisms and pathways at play. This review aims to give a narrative summary of the literature on celiac disease's neurological symptoms, particularly migraines, and to assess any potential associations to dysbiosis, an imbalance in the microbiome.

## Introduction and background

Approximately 1% of the global population suffers from celiac disease, an autoimmune reaction to gluten caused by a hereditary predisposition. It is characterized by intraepithelial lymphocytic infiltration, villous atrophy, and crypt hyperplasia. A diverse range of clinical manifestations can be seen, from gastrointestinal (GI) to neurological symptoms [1]. The most common GI presentations are abdominal pain, bloating, constipation, diarrhea, failure to thrive/weight loss, nausea, reflux, and vomiting. Extraintestinal manifestations include abnormal liver enzymes, arthralgia/arthritis, dermatitis herpetiformis, alopecia, fatigue, anemia, stomatitis, myalgia, psychiatric disorders, rashes, short stature, delayed puberty, osteoporosis, and infertility [2]. Celiac disease manifests neurologically in many ways, including epilepsy, ataxia, mood disorders, encephalitis, peripheral neuropathy, neuromuscular disorders, dementia, learning disorders, and developmental delay [3]. Among all presentations, migraine represents one of the main features of celiac disease [4].

According to the International Classification of Headache Disorders, migraine is a debilitating condition involving moderate to severe headache episodes that last four to 72 hours and are accompanied by nausea or vomiting. Over 17% of women are affected, while 5% to 8% of men are affected [5]. In addition, it is linked to various GI diseases demonstrating a strong association between neurological and gastrointestinal presentations. This further implies that migraines may be connected to abnormalities in the gut-brain axis [6]. 

Although the exact pathophysiology of migraine is not fully understood, the serum of individuals who suffer from migraine contains elevated amounts of pro-inflammatory cytokines such as tumor necrosis factor-alpha (TNF-α) and interleukin (IL)-1 [1]. Calcitonin gene-related peptide (CGRP), which has been demonstrated to be secreted during migraine attacks, is found in over half of the neurons in the trigeminal ganglion, the region where these cytokines exercise their influence. Trigeminovascular activation that is repeated and persistent can sensitize brainstem nuclei, leading to a state of central sensitization. In a nutshell, it is believed that the pathophysiology of migraine is mediated by the sequential events of neurogenic inflammation, peripheral trigeminovascular input, and central cortico-trigeminal nuclei activation [5]. In celiac disease, the immune system overreacts to gluten, resulting in a T-cell-mediated autoimmune enteropathy that damages the small intestine [7]. The inflammatory response leads to the activation of enteric neurons and the subsequent release of cytokines [8]. One theory is that rather than being directly antibody-mediated, neurological manifestations in celiac disease may be brought on by a systemic inflammatory response. Thus, the elevated levels of interferon-gamma and TNF-α, which regulate the synthesis of CGRP, could account for the link between migraine and celiac disease [5].

The neuropeptide is also considered to have an antimicrobial effect on a range of gut bacterial strains (including *Escherichia coli*, *Enterococcus faecalis*, and *Lactobacillus acidophilus*) [3]. Furthermore, the presence or absence of gluten in the diet changes the diversity and number of microbial populations in the gut microbiota, potentially producing dysbiosis [9]. By affecting the gut-brain relationship, the gut microbiota may have a role in neurological diseases, including migraine [10]. This review article discusses the impact of celiac disease and changes in gut microbiota on migraine development.

Search strategy

We performed a title search in PubMed, PubMed Central, and Medline using the following keywords: celiac disease and migraine, microbiota and migraine, gluten and microbiota, celiac disease and microbiota, and gut-brain axis. All the articles considered were chosen without the restriction of time of publication or study type, i.e., traditional reviews, systematic reviews, clinical trials, case-control studies, and cohort studies. Age and ethnicity were not factored in in the studies. There were no restrictions on the search based on demographics. All the articles chosen were in the English language.

## Review

In this discussion, we will review the existing knowledge on the functional composition and homeostasis of the gut microbiota, the intestinal microbiota, and celiac disease; interactions between the GI system and the central nervous system (CNS); and a detailed description of the gut pathways involved in the development of migraines.

Gut microbiota

'Microbiome' is the term used to describe the entire genome of the human microbiota [11]. A diverse ecology is maintained by this abundant microbiota population, which is thought to number 100 trillion microorganisms [12]. About 10 times as many bacteria as human cells are present in an adult's body, with 80% of them being in the gut. More than 5000 microbe strains and more than 1000 different types of microflora are known to make up the human microbiome [13,14]. Bacteria, primarily anaerobic bacteria, dominate this habitat. However, viruses, protozoa, archaea, and fungus are also present [15,16]. It is primarily defined by two bacterial phylotypes, Bacteroidetes andFirmicutes, with minor contributions from Proteobacteria, Actinomyces, Fusobacterium, and Verrucomicrobia [17].

The gut microbiota serves several purposes. First, the intestinal microbiota forms the intestinal barrier, promotes the intestinal microbiota's survival, stimulates intestinal epithelial cells' regeneration, produces mucus, and nourishes the mucosa by producing short-chain fatty acids (SCFAs) [18]. It is also involved in developing the immune system by stimulating the innate immune system at an early age, leading to the maturation of gut-associated lymphoid tissue and the activation of adaptive immunity by promoting local and systemic immune responses [19]. In addition, it promotes the intestinal synthesis and metabolism of certain nutrients, hormones, and vitamins and plays a vital role in drug and poison removal. A state of low degree of physiological inflammation, an efficient defense against microorganisms, results from gut microbiota continuing to stimulate the immune system under physiological settings [20]. Furthermore, bacterial colonies play a defensive competition role in the gut by producing cytokines that can hinder the growth of the microorganism and nutrition for the pathogens' survival [21].

Human development and numerous stressors impact how the gut flora changes. Mothers provide babies with the foundational microbiota [22]. Infants have a diverse gut flora similar to adults after becoming one year old [23,24]. The gut microbiota's makeup is not constant and evolves as people age. Although the dynamic alterations vary significantly between individuals, a macro equilibrium is a general outcome [25]. While some factors, like autoimmune disease, infection, drugs, illness, and food, may influence the microbiome, beneficial bacteria changes can substantially impact an individual's health [26,27].

Gut microbiota and gluten

In Western countries, gluten is a necessary nutritional ingredient. Prolamins, which include gliadin and glutenin, is a complex blend of proteins that make up gluten [9]. Its consumption has significantly expanded over the past century, and with it, celiac disease prevalence has significantly increased. When gluten is not entirely degraded, immunogenic peptides are produced and interact with the intestinal epithelium and mucosa [28]. In humans, the duodenal microbiota and gluten metabolism are closely linked [29]. Certain microorganisms may be in charge of some of the observed proteolytic activity, according to specific patterns of gliadin breakdown in duodenal biopsies of celiac disease patients that have been characterized [30]. Although the small intestine has lower microbial diversity than the large intestine, the high luminal concentration of gluten proteins encourages the growth of proteolytic bacteria [31].

Due to the substantial gluten consumption in Western countries, the small intestine has many gluten peptides. These peptides serve as a food source for various duodenal bacteria that cause dysbiosis in people with celiac disease [30]. The number of bacterial species, diversity, and proportions is altered in celiac disease due to the activation of the immunological cascade that causes the inflammatory response [32,33].

Gut-brain axis

A bidirectional interaction between the GI system and the CNS is indicated by the term 'gut-brain axis.' The brain controls the GI tract's movements and operations. The effects of hormonal variables on gut functioning are mediated by the hypothalamus pituitary adrenal (HPA) axis, which mediates stress responses. Conversely, it is thought that the GI system can impact the CNS. The gut system influences a range of brain processes, including cognition, behavior, and even nociception. Numerous neurological conditions, including migraines, have been tied to the disruption of the gut-brain axis [34,35].

Gut microbiota and migraine

**Figure 1:**
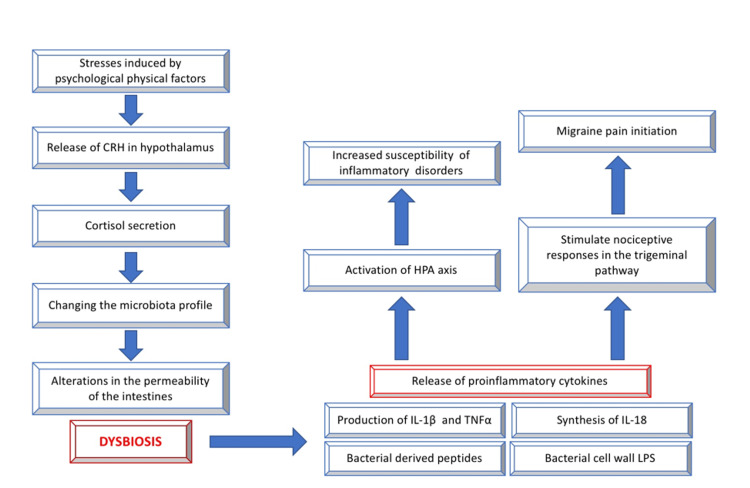
Mechanisms by which gut bacteria maintain a healthy balance in the gut-brain axis Hodan Qasim, the first author, created this figure. The paper *Gut-brain Axis and migraine headache: a comprehensive review *[3]​​,* *an article distributed under the terms of the Creative Commons Attribution 4.0 International License, served as the source for the figure. CGRP: Calcitonin gene-related peptide, CRH: Corticotrophin-releasing hormone, HPA: Hypothalamic pituitary adrenal axis, LPS: Lipopolysaccharides, IL: Interleukin, TNFα: Tumor necrosis factor-alpha

Migraines are among the neurological illnesses that the gut microbiome can influence through gut-brain communication. According to some data, the gut microbiota can modulate the brain-gut axis through various routes, which may impact nociceptive behavior and brain function [34]. A layer of columnar intestinal epithelial cells separates the 100 trillion bacteria present on the gut surface from the host. The key pathophysiological processes connected to migraine are thought to work partly due to the gut microbiota composition, which also plays a significant role in the gut-brain axis [35]. Neurotransmitters, hormones, and inflammatory chemicals originating from the microbiome have all been identified as pathways. Figure 1 below outlines the indirect connections and the role of dysbiosis [3].

Neurotransmitters

Serotonin, gamma-aminobutyric acid, and CGRP have all been involved in this process [35-37]. Gut bacteria can produce these neurotransmitters, and these compounds can be exchanged between various cell types [38]. In particular, intestinal cells in the gut can produce large amounts of 5-hydroxytryptamine (5-HT), impacting the neurological system [39-40]. As a result, the human gut microbiota produces many essential neurotransmitters that impact the body, particularly on the gut-brain axis [41].

Table 1 summarizes some studies highlighting the association between neurotransmitters and gut dysbiosis.

**Table 1:** Literature on neurotransmitters and gut dysbiosis CGRP: Calcitonin gene-related peptide, GABA: Gamma-aminobutyric acid, CSF: Cerebrospinal fluid, GI: Gastrointestinal

Neurotransmitter	Results
Serotonin	Tryptophan, tyrosine, and glutamine levels in the whole brain are lower in germ-free mice than in mice recolonized by normal microbiota [42], whereas 5-HT and 5-hydroxyindoleacetic acid concentrations in hippocampal slices are higher in germ-free mice [43]. Additionally, when compared to animals with normal gut microbiota, germ-free animals have higher levels of 5-HT and tyrosine in their blood [43,44].
CGRP	Studies in animal models showed that peripherally administered CGRP, one of the primary biomarkers of migraine, suppresses gastric acid secretion [45]. Additionally, by altering a central vagal outflow, CGRP is a powerful inhibitor of pancreatic enzyme secretion [46].
GABA	Previous studies have shown that migraineurs' plasma and CSF levels of glutamate are higher than those of control patients [47,48]. This neurotransmitter has a well-established function in the gut-brain axis, which involves the transmission of afferent neurons from the gut to the brain. Additionally, it was suggested that glutamate might impact the GI tract's oxidative stress and inflammation [49].

Hormones

Through the sympathetic and parasympathetic nervous systems and by producing neuroendocrine peptides, the CNS can influence the gut flora [50]. Stress-related variables on the physical and mental levels can alter the microbial makeup of the gut. These stressors cause the hypothalamus to release a corticotrophin-releasing hormone, which triggers the adrenal glands to release cortisol. Additionally, these stressors may change the microbiota profile, altering the permeability of the intestines and resulting in dysbiosis [51,52].

Inflammatory Molecules

Immune cells and their inflammatory mediators, such as IL-1, IL-6, IL-18, TNF- α, and interferon‐gamma (IFN-γ), have been shown to sensitize afferent terminals and are known to elicit visceral pain in the gut [53]. Pro-inflammatory cytokines such as IL-1, IL-6, IL-8, and TNF-α, have also been linked to migraine pain and are elevated during episodes [54]. Inflammation and gut permeability are correlated in a bidirectional manner, thus increased gut permeability can trigger inflammatory and immunological responses through lipopolysaccharide (LPS) leakage, and pro-inflammatory cytokines can then increase gut permeability [55]. However, gluten may also impact nociceptive responses in the trigeminal pathway and contribute to the onset of migraine pain. These pro-inflammatory cytokines include TNF-α, IL-1, and IL-6 [56-60].

Limitations

However, this study has some limitations, and there are a number of remaining questions. The search strategy for this article review was limited to three databases i.e., PubMed, PubMed Central (PMC), and Medline. Theoretically, it is unclear if the gut microbiota has any impact on large-scale brain function. Dysbiosis is evident in celiac disease, but it is still unclear whether this is a cause or a result of it, and whether celiac disease has a distinct pattern of change. Therefore, more research is required to determine the true effects of dysbiosis and to characterize the pathways involved in celiac disease-related migraines, making this an intriguing area for further study.

## Conclusions

The relationship between celiac disease and migraine is significantly influenced by the gut-brain axis. The primary cause of the significant involvement of many inflammatory and vasoactive mediators in the physiopathology of migraine is the regulation of the GI immunological and autonomic system by the GI microbiota. Even though the mechanism underlying this relationship is not entirely known, the available research suggests that the gut-brain axis may affect migraines. In general, various factors, including the composition of the gut microbiota, dietary components, neurotransmitters, hormone pathways, and inflammatory mediators (IL-1, IL-6, IL-8, and TNF-α), influence this interaction. Since some gut bacterial strains are known to be negatively impacted by neuropeptides, including CGRP, it has been hypothesized that these neuropeptides are also involved in the reciprocal communication between the gut and the brain. Although knowledge has tremendously advanced, it is still unclear how higher gluten exposure, immune system alterations, and disruptions in the gut microbiota may interact to cause migraine headaches in people with celiac disease.
